# Whole Genomic analysis of a clinical isolate of Uropathogenic Escherichia coli strain of Sequence Type - 101 carrying the drug resistance NDM-7 in IncX3 plasmid

**DOI:** 10.6026/97320630017126

**Published:** 2021-01-31

**Authors:** Venkatesan Ramakrishnan, Xavier Alexander Marialouis, Jagadesan Sankarasubramanian, Amutha Santhanam, Anand Setty Balakrishnan

**Affiliations:** 1 Department of Genetic Engineering, School of Biotechnology, Madurai Kamaraj University, Madurai 625021, Tamil Nadu, India; 2 National Centre for Nanoscience and Nanotechnology, University of Madras, Guindy Campus, Chennai 600025, Tamil Nadu, India; 3 National Institute of Pharmaceutical Education and Research, 168, Manicktala Main Road, Kolkata 700054, West Bengal, India; 4 Department of Genetics, School of Biological Sciences, Madurai Kamaraj University, Madurai 625021, Tamil Nadu, India

**Keywords:** Genomic analysis, Escherichia coli, isolate, drug resistance

## Abstract

The emerging NDM-producing Enterobactereciae is a major threat to public health. The association of NDM-7 with sequence type 101 E.coli is identified in very few numbers. Therefore, it is of interest to analyse the whole genome sequence of NDM-producing uropathogenic E. coli XA31 that was found to carry numerous drug resistance genes of different antibiotic classes. The isolate E. coli belongs to ST-101 carrying blaNDM-7 coexisting with several resistance genes blaOXA-1, blaTEM1-A, blaCTX-M15, aac(6')-Ib-cr, catB3, tetB. Resfinder predicts this and four other plasmid replicons were identified using the Plasfinder in the CGE platform. The high transferable IncX3 plasmid was found to carry the NDM-7 gene. Thus, we the report the combination of NDM-7-ST101-IncX3 in India. The combination of this epidemic clone with NDM-7 is highly required to develop an effective infection control strategy.

## Background:

The majority of community-acquired and nosocomial spread of Urinary tract infection (UTI) is highly mediated by the uropathogenic Escherichia coli (UPEC) in humans. This mostly affects the women, among half of them experience this at least once in their lifetime [1]. Though the Escherichia coli is the major determinant of urinary tract infection it also involve in causing other diseases including bacteraemia and liver abscesses [2]. These bacterial strains develop resistance to β-lactam antibiotics through conferring ESBL [3]. To overcome that, Carbapenems the last line drug is being in practice for a long time to handle the ESBL producers of Enterobactereciae [4], since its extensive use on the ESBL producers has lead to the emergence of carbapenemases that hydrolyses all β lactam including carbapenems [2,3]. Carbapenemases are a broad category of β-lactamases based on their functional properties that fall in different Ampler classes A B D. NmcA serine beta-lactamases, chromosomally encoded Sme, IMI-1, and SFC-1 and plasmid-encoded KPC enzymes come under carbapenemase class A. VIM types, IMP, GIM-1, SPM-1, and SIM types of enzymes and NDM-1 carbapenemase grouped in class B. And OXA-48 comes under class D [5]. Among them, the increasing incidence of New Delhi Metallo β lactamase (NDM) has to lead to widespread detection of many variants causing serious infection [6]. Recently a new paradigm was developed through preclinical treatment as a new compound ANT 2681 combine with meropenam to overcome serious infection of NDM producing Enterobacteriaceae [7]. Of these NDM variants, the majority of them have successfully disseminating on most of the Enterobacteriaceae by mobilizing into various plasmid incompatible groups like IncX3, IncA/C2, and IncF, IncH, and IncL/M [6], through different molecular evolutionary events like horizontal gene transfer and others [8]. This results in the global prevalence of major NDM variants such as NDM-1, NDM-5, NDM-7 [3]. Moreover, the association of these NDM variants with high transferability plasmids like IncX3 in possibly emerging epidemic sequence type 101 E. coli is also reported [9,10]. Therefore, it is of interest to analyse the whole genome sequence of NDM-producing uropathogenic E. coli XA31 that was found to carry numerous drug resistance genes of different antibiotic classes.

## Methodology

### Bacterial strains and antibiotic susceptibility:

A total of 54 isolate was collected in the year 2011 from various patients treated with urinary tract infection and processed for their resistance against various antibiotics except for carbapenem in the previous study [11]. Only two strains among the others have shown resistance to various antibiotics with imipenem and meropenem, the strain XA31 with extra resistance to tetracycline was selected for the whole genome sequencing.

### Detection of carbapenemase:

To detect carbapenemase enzyme, the double synergy disc containing the combination of Ethylenediaminetetra acetic acid (EDTA) with imipenem/meropenem antibiotics were placed separately on E. coli XA 31 broth culture swabbed LB agar plate (data not included).

### Detection of NDM on plasmid:

The plasmid DNA was isolated by both alkaline lysis and Qiagen kit method. Using total plasmid as the template the NDM gene was amplified by PCR with gene-specific primer [12]. Further, the different band of plasmid DNA on agarose gel was eluted using the Qiagen kit to identify the NDM gene carrying specific plasmid by PCR using an NDM-specific primer. The resulting amplicons were separated by 0.8 %(w/v) agarose gel stained with ethidium bromide and viewed by UV transilluminator.

### Whole-genome sequencing:

The genomic DNA and plasmid DNA of E. coli strain XA31 were isolated by the C-TAB method. The library preparation with 2 x 150 bp chemistry and sequencing was carried out by Illumina NextSeq500 platform according to the manufacturer instructions.

### Bioinformatics analysis:

The resulting library of raw sequence reads was assembled by Spades version 3.12.0 [13]. And the cut-off of 500bp was set using QUAST [14] to filter poor reads as below 500 BP and was discarded manually. The 138 assembled contigs were annotated by RAST (http://rast.nmpdr.org/) [15], Prokka [16], and NCBI prokaryotic genome automatic annotation pipeline (PGAAP). (http://www.ncbi.nlm.nih.gov/genomes/static/Pipeline.html) [17]. The Sequence type and phylogroup were identified by actman7 gene MLST in the Entrobase database [18]. Plasmid replicon and resistance genes were identified by Res Finder and Plasmid finder respectively in the CGE server [19,20] (https://cge.cbs.dtu.dk/services/). Further, plasmid sequence from the whole genome was separated by PlasmidSpades [21], and separated plasmid sequences were mapped by r2cat mapping tool [22] against the respective reference plasmids and subsequent pairwise BLAST alignment available at NCBI (https://blast.ncbi.nlm.nih.gov/Blast.cgi?) Finally, the whole genome and the plasmids graphical image were generated by the CGview server [23] and the genetic environment of NDM-7 was analyzed by Easy fig 2.2.5 [24].

## Results:

### Phenotypic and genotypic characterization:

The UPEC strain XA31 was resistant to numerous antibiotics such as ampicillin, amoxiclav, imipenem, meropenem, cefoxitin, ceftriaxone, cefixime, cefepime, cefotaxime, ceftazidime, ceftazidime/clavulanic acid, gentamicin, nalidixic acid, ciprofloxacin, and susceptible to cotrimoxazole, nitrofurantoin. The resistance phenotype and the Metallo β lactamase production were identified by EDTA/imipenem synergistic activity. Based on the result, the gene encoding NDM was randomly identified using a specific primer by PCR. Further, to detect the NDM gene on the plasmid DNA of E. coli XA31, the total plasmid DNA was used as a template in which the gene encoding NDM was identified by PCR. And it was required to find the NDM, TEM, CTX-M-15 genes carrying specific plasmid on total plasmids; each separate plasmid fraction of the total plasmid was used as individual template DNA, among them, the above resistance genes were identified in an uncharacterized plasmid that positioned in front order of agarose gel. Due to its low copy number, the single plasmid fraction could not be characterized by sequencing.

### Molecular characterization:

Whole-genome sequencing was completed to characterize the antimicrobial resistance genes and their corresponding conferring plasmid on E. coli XA31. As a result of sequencing and annotation, the 5.2 MB of the genome was obtained with various molecular parameters. Also various resistance genes particularly the NDM as NDM-7 that corresponds to the respective antibiotic phenotypic result and few non-phenotypical identified resistance genes importantly OXA-1 were identified in the Whole genome sequencing (WGS) by Resfinder. Moreover, plasmid finder identified four types of plasmids in WGS. Further, the whole genome was identified as sequence type (ST) 101 by Achtman MLST and deposited in entrobase with the barcode of ESC_OA2793.

### Separation of IncX3 plasmid conferring NDM-7:

The total plasmids of WGS were separated through plasmid Spades assembly. The resulting plasmid sequences were mapped against BLAST hits generated with respective reference plasmids by r2cat. Once again the sequences of plasmids were confirmed by doing pairwise alignment BLAST with the references. Moreover, the replicon of each segregated plasmid was identified as 1.3 KB col1 the bacteriocin plasmid, 132.8 KB Inc1 plasmid, 167.6 KB IncC plasmid, and 41.6 KB IncX3 plasmid-by-plasmid finder. Finally, Resfinder screened all the four plasmids for the NDM-7 against BLAST hits with respective reference. As a result, the presence of the NDM-7 gene only in the Resfinder result page of IncX3 plasmid and not in the rest of the plasmids confirms the 41.6KB IncX3 plasmid carrying the gene NDM-7.

### Characterization of the genetic environment of NDM-7:

The plasmid annotated by Prokka was compared with the following IncX3 reference plasmids pKW53T-NDM (KX214669), pKpN01-NDM7 (CP012990), pEC25_NDM-7(CP035125), pJN05NDM7 (MH523639), pM110_X3 (AP018141), pABC133-NDM(KX214671) carrying NDM-7 by BLAST analysis. The result shows that the NDM-7 is flanked by the IS6 family of transposase, Tn3 family of transposase in the downstream region; bleomycin resistance protein, Indol-3-glycerol-phosphate synthase, Thiol: disulfide interchange protein (DsbD), and hypothetical protein in the upstream region of the genetic environment in the pXA31 IncX3 plasmid. The type of transposable elements in pXA31 has differed from the transposable elements carried by different IncX3 reference plasmids.

## Discussion:

The presence of NDM-7 with OXA-1 and numerous antibiotic resistance genes carrying sequence type (ST) 101 E. coli associated with urinary tract infection is reported from this study. The NDM trait has been spreading worldwide since its first detection from a Swedish patient [28]. Over the world, the prevalence rate of NDM is higher in Asia (82%) particularly in China and India [4,9]. To date, there are 29 different NDM types have been identified (https://www.ncbi.nlm.nih.gov/pathogens/refgene/#gene_family:(blaNDM)), among them the widespread existence of some NDM alleles majorly NDM-1 and followed by NDM-5, NDM-7, and NDM-4 has been reported worldwide [3]. There have been many reports of NDM-7 associating with plasmids IncA/C, IncF, and IncX3 since its first report from 2013 [26,27,28,29, 30]. Particularly most of the NDM variants have been found with IncX3 plasmid type due to its high transferability of nature, also this plasmid was found in different species [9,28, 29,31,32,33,34]. Even though in India already reports were stating that IncX3 mediated NDM-7 in sequence type-167 and NDM-1 on other plasmid carried by ST 101 E. coli.[35,36]. In addition to this, many reports of E. coli isolates in India found with NDM-7 in IncX3 and other plasmid types was not yet identified the sequence types as well that results in the lack of information about the prevalence of E. coli with respective ST [26,37]. Only two findings are available about the incidence of the NDM-7/ST101 association, which are one in Europe and another in Asia. According to previous reports avilable [26,36]. The combination of ST 101 E. coli-IncX3-NDM-7 was observed. But in globally various reports state that NDM producing E. coli from clinical samples is mostly identified as ST101, epidemic spread of this ST101/NDM-1 combination found in most of the countries [4, 10,36]. To analyze the genetic environment of NDM-7 in IncX3 plasmid of strain XA31, we compared it with five different IncX3 plasmids. From the comparisons, it reveals that the pXA31IncX3 plasmid genetic environment composed of NDM-7 that positioned between bleomycin resistance protein and Tn3 family transposase and module of genes that surrounds the NDM-7 is mostly same as like in reference plasmids but it solely differs in the kind of Tn3 family and IS6 family of transposase in the downstream region that it carries. The association of another resistance marker the bleomycin resistance protein upstream of NDM-7 has been found on most of the NDM variants denotes that both of them mobilized to form a common progenitor to protect the NDM [38,39]. And the presence of Tn3 family IS3000 downstream of NDM-7 in this IncX3 plasmid and also in different plasmids in India indicates that the Tn3 family transposase is an ancestral structure, and the rate of mutation is likely very less as reported by Zhao et al [40]. Hence, it suggests that with the stable Tn3 family transposase and selection marker bleomycin resistance protein the NDM-7. Its variants are possibly disseminating through the higher transferring efficiency plasmid IncX3 on different species. "Genome Data" The whole genome was deposited under the Bioproject PRJNA483269 in NCBI/DDBJ/ENA with the Accession number of QRBD00000000.

## Conclusion

It is necessary to develop effective strategies in infection management to control and prevent the spreading of the NDM variants using different plasmids particularly IncX3 on different species to reduce the risk of emerging resistance to the last line drugs in therapeutics.
